# Four-year effectiveness, safety and drug retention rate of secukinumab in psoriatic arthritis: a real-life Italian multicenter cohort

**DOI:** 10.1186/s13075-024-03401-x

**Published:** 2024-09-28

**Authors:** Roberta Ramonda, Mariagrazia Lorenzin, Maria Sole Chimenti, Fabiola Atzeni, Angelo Semeraro, Salvatore D’Angelo, Carlo Selmi, Augusta Ortolan, Antonio Marchesoni, Maria Manara, Michele Maria Luchetti Gentiloni, Leonardo Santo, Carlo Salvarani, Alberto Cauli, Maurizio Rossini, Giorgio Amato, Giacomo Cozzi, Laura Scagnellato, Mario Ferraioli, Antonio Carriero, Elena Fracassi, Francesco Giorgio, Andrea Doria, Rosario Foti, Antonio Carletto, on behalf Spondyloarthritis and Psoriatic Arthritis SIR Study Group “Antonio Spadaro”, Roberta Ramonda, Mariagrazia Lorenzin, Maria Sole Chimenti, Fabiola Atzeni, Angelo Semeraro, Salvatore D’Angelo, Carlo Selmi, Augusta Ortolan, Antonio Marchesoni, Maria Manara, Michele Maria Luchetti Gentiloni, Leonardo Santo, Carlo Salvarani, Alberto Cauli, Maurizio Rossini, Giorgio Amato, Laura Scagnellato, Mario Ferraioli, Elena Fracassi, Francesco Giorgio, Andrea Doria, Rosario Foti, Antonio Carletto, Roberta Foti, Elisa Visalli, Ylenia Dal Bosco, De Lucia Francesco, Cesaro Siracusano, Sergio Collela, Nicoletta Luciano, Valentino Paci, Giulia Marchionni, Nicolò Girolimetto, Alberto Floris, Giorgia Citriniti, Giovanni Striani, Antonio Carriero, Roberta Foti, Elisa Visalli, Ylenia Dal Bosco, De Lucia Francesco, Cesaro Siracusano, Sergio Collela, Giacomo M. Guidelli, Nicoletta Luciano, Valentino Paci, Giulia Marchionni, Nicolò Girolimetto, Alberto Floris, Giorgia Citriniti, Giovanni Striani

**Affiliations:** 1 Department of Medicine DIMED, Rheumatology Unit, University of Padova, Padova, Veneto Italy; 2 Department of Systems Medicine, Rheumatology, Allergology and Clinical Immunology, University of Rome Tor Vergata, Rome, Lazio Italy; 3 Rheumatology Unit, University of Messina, Messina, Sicilia Italy; 4 Rheumatology Unit, Martina Franca-ASL Taranto, Martina Franca Hospital, Martina Franca, Taranto, Puglia Italy; 5 Department of Health Sciences, University of Basilicata, Potenza, Basilicata, Italy; 6 Rheumatology and Clinical Immunology IRCCS, Humanitas Research Hospital, Milan, Lombardia Italy; 7 Department of Biomedical Sciences, Humanitas University, Milan, Lombardia Italy; 8 Rheumatology Unit, Fondazione Policlinico Universitario Agostino Gemelli IRCSS, Rome, Lazio Italy; 9 Rheumatology, Humanitas San Pio X, Milan, Lombardia Italy; 10 Department of Rheumatology, ASST Gaetano Pini-CTO, Milan, Lombardia Italy; 11 Department of Clinical and Molecular Sciences, University Hospital of the Marche Region, Ancona, Marche, Italy; 12 Rheumatology Unit, ASL BT Andria – DSS4 Barletta, Italy, Barletta-Andria-Trani, Puglia, Italy; 13 Rheumatology Unit, Department of Internal Medicine, Azienda USL-IRCCS, University of Modena and Reggio Emilia, Emilia Romagna, Italy; 14 Rheumatology Unit, Department of Medical Sciences, AOU and University of Cagliari, Monserrato, Sardegna Italy; 15 Rheumatology Unit, Department of Medicine, AOUI University of Verona, Verona, Veneto Italy; 16 Rheumatology Unit, A.O.U. Policlinico S. Marco, Catania, Sicilia, Catania, Italy; 17 Rheumatology Unit, Department of Medicine-DIMED, Padova University Hospital, Via Giustiniani, 2, 35128 Padova, Italy; 18 Rheumatology Institute of Lucania (IReL), San Carlo Hospital of Potenza and Madonna delle Grazie Hospital of Matera, Potenza, Basilicata, Italy

**Keywords:** Psoriatic arthritis, Biologics, Secukinumab, Remission/Effectiveness, Safety, Drug retention rate

## Abstract

**Objectives:**

to evaluate over a 48-month follow-up period the: 1) long-term effectiveness and safety; 2) drug retention rate (DRR); 3) impact of comorbidities and bDMARDs line on MDA and DAPSA remission/low disease activity (LDA) of secukinumab in a multicenter Italian cohort of PsA patients.

**Methods:**

Consecutive PsA patients receiving secukinumab were followed prospectively in Italian centers between 2016 and 2023. Disease characteristics, previous/ongoing treatments, comorbidities and follow-up duration were recorded. Treatment response was evaluated at 6 and 12 months after initiation, and every year up to 48 months (T48). DRR was assessed according to clinical and demographic features, comorbidities and bDMARDs line. Adverse events (AE) were recorded.

**Results:**

Six hundred eighty-five patients [42.5% male] were enrolled; 32.9% naïve received secukinumab; 74.2% had ≥ 1 comorbidity. Overall, secukinumab yielded improved outcomes at T48: naïve maintained lower disease activity vs. non-naïve [DAPSA 4.0 (1.4–8.1) vs. 6.0 (2.2–10.4);*p* = 0.04]; 76.9% naïve and 66.2% non-naïve achieved MDA; MDA no comorbidities vs. 1–3 comorbidities 78.8% vs. 73.3% (*p* < 0.05), and MDA no comorbidities vs. > 3 comorbidities 78.8% vs. 48.7% (*p* < 0.001). DAPSA-REM and DAPSA-LDA rates were higher in naïve patients, albeit similar between those without comorbidities vs. 1–3 comorbidities, and slightly lower in those with > 3 comorbidities. Treatment was discontinued in 233 patients due to loss of effectiveness, and in 41 due to AE. The overall DRR at T48 was 66%, with differences according to bDMARDs line (*p* < 0.001), use of combined csDMARDs (*p* = 0.016), BMI (*p* = 0.037) and mono/oligoarthritis vs. polyarthritis (*p* = 0.012).

**Conclusions:**

Secukinumab proved safe and effective, and patients achieved sustained remission with a notable drug retention rate at 4 years.

**Supplementary Information:**

The online version contains supplementary material available at 10.1186/s13075-024-03401-x.

## Key messages

***What is already known on this topic?***

• Secukinumab is a novel drug for psoriatic arthritis (PsA), but real-life long-term safety and effectiveness data are lacking.

***What this study adds?***

• Our findings confirmed the safety and notable effectiveness on all PsA domains (arthritis, enthesitis, dactylitis, spinal symptoms, psoriasis, PROs and inflammatory markers), over a 48-month follow-up period.

• The drug retention rate (DRR) is considerably high at 48 months. The main clinical disease pattern (peripheral/axial involvement), male gender, age, and the presence of comorbidities do not influence the DRR of secukinumab over time.

• The first line of bDMARDs seems to favor MDA and remission/low disease activity DAPSA achievement and drug retention, while fewer than 3 comorbidities have no impact on these outcomes.

***How this study might affect research, practice or policy***

• This study supports the effectiveness of secukinumab, which also seems to be a valid option for multi-drug failure patients; the safety of secukinumab means it can be used in patients with comorbidities, older age, higher BMI, and in particular cardiovascular conditions and metabolic syndrome.

**Supplementary Information:**

The online version contains supplementary material available at 10.1186/s13075-024-03401-x.

## Introduction

Psoriatic arthritis (PsA) is a chronic, inflammatory disease characterized by widespread musculoskeletal manifestations in patients with psoriasis [1, 2]. PsA is characterized by a heterogeneous clinical presentation and different courses of the disease [3].

Improved understanding of the pathogenesis of PsA has led to the development of biologic medications and small molecules targeting specific cytokines and signaling pathways, which have been shown to prevent disease progression and improve quality of life [4, 5]. These biologic agents are recommended for the treatment of active moderate-severe PsA in adults with inadequate response to previous conventional synthetic disease modifying anti-rheumatic drugs (csDMARDs) [6–8].

Secukinumab is a human monoclonal antibody (IgG1) that targets IL-17A, approved for the treatment of plaque psoriasis (PsO) [9], PsA and axial spondyloarthritis (AxSpA) [10, 11]. Secukinumab has shown efficacy and safety in biologic-naïve patients with PsA and in those previously exposed to anti-tumor necrosis factor (TNF) inhibitors (FUTURE-1 and FUTURE-2) [10, 12], but it has not demonstrated superiority to adalimumab (EXCEED) [13]. Secukinumab has also demonstrated rapid and sustained improvement in signs, symptoms, physical function, and improvement of health-related quality of life (HRQoL) in patients with PsA over 5 years across the Phase 3 FUTURE trial [14]. Data from randomized controlled trials (RCTs) and post-marketing surveillance have shown that secukinumab has a favorable safety profile over long-term treatment [15], with fewer adverse events and a low treatment discontinuation rate [16–18].

In this clinical heterogeneity scenario, with a broad arsenal of treatments available, clinicians need to demand better results in terms of effectiveness and safety beyond the information available predominantly from multiple RCTs. The strict enrolment criteria of RCTs may limit the extrapolation of the results since trial-selected cohorts are often not fully representative of the patients encountered in daily clinical practice who may have multiple comorbidities or other clinical features influencing the management and treatment response [19, 20].

In the context of PsA, few studies have investigated the effectiveness and safety of secukinumab in a real-life setting both in Italian [21–23] and international cohorts [24–26], but only for a limited observational period. In addition, the impact of more lines of prior biologic (b) DMARDs, comorbidities, and clinical features on the achievement of clinical remission and on secukinumab drug survival has not yet been fully investigated [27–33].

This prospective observational study aimed to evaluate, in a multicenter, Italian, real-life cohort of PsA patients on secukinumab, followed up for 48 months: 1) long-term effectiveness and safety; 2) the drug retention-rate (DRR) and reasons for discontinuation; 3) the impact of comorbidities and previous bDMARD treatment lines on achieving minimal disease activity (MDA) and Disease Activity in Psoriatic Arthritis (DAPSA) remission/low disease activity.

## Material and methods

### Study design, patients and data source

This is an observational study based on a prospectively recorded database of patients with PsA treated with secukinumab from September 2016 to May 2023 in 15 Italian Rheumatology centers. The study was supported by the Italian Society of Rheumatology’s (SIR) “Spondyloarthritis and Psoriatic Arthritis study group—A. Spadaro”.

The study was conducted in compliance with the principles of the Declaration of Helsinki, when they were first entered into the database for treatment. The Ethics committee’s approval was obtained from all participating centers [approval no. 23943], as well as written informed consent for the anonymous use of personal data from every patient, in compliance with Italian Legislative Decree 196/2003.

Demographic patient characteristics (age, gender, body mass index [BMI]), disease characteristics, clinical presentation as axial, peripheral, or mixed, other clinical manifestations (i.e. enthesitis, dactylitis, skin and/or nail involvement, extra-articular manifestations), disease duration, diagnosis age, previous/ongoing treatments, concomitant medications, including conventional synthetic disease-modifying antirheumatic drugs (csDMARDs), or non-steroidal anti-inflammatory drugs (NSAIDs), or glucocorticosteroids (GCs), or previous biologics were collected when secukinumab was administered for the first time. Due to an expected difference in treatment retention and response between naïve patients and those previously treated with one or more biologic (b) DMARDs, information was collected about the first and second (or more) treatment line before secukinumab treatment. Any comorbidities were also recorded and defined as coexisting medical conditions distinct from the principal diagnosis for which the patient was included in this study. Baseline data were retrieved by reviewing the clinical charts, face-to-face interview, and extensive patients’ medical record.

### Patients and follow-up

Patients diagnosed with PsA according to the ClASsification for Psoriatic ARthritis (CASPAR) criteria [34], and who initiated secukinumab for moderate or severe disease according to the European Alliance of Associations for Rheumatology (EULAR) and/or the Group for Research and Assessment of Psoriasis and Psoriatic Arthritis (GRAPPA), and/or the Italian Society of Rheumatology (SIR) guidelines were considered, [6–8] and those who persisted with the treatment for more than 3 months were included. Patients underwent a series of screening tests before enrolment and starting treatment, as recommended by the European guidelines [6–8]. Secukinumab was administered subcutaneously at a dosage of 150 mg or 300 mg as needed – at the discretion of the treating rheumatologist – for PsO or multi-drug failure at weeks 0, 1, 2, 3, 4, and every 4 weeks thereafter in accordance with the manufacturer’ instructions [35]. Follow-up started at the treatment initiation date of secukinumab and ended at the treatment discontinuation date, death, or the end of the study (31 May 2023), whichever occurred first. Finally, the duration of secukinumab treatment expressed in months, lines of bDMARDs, reasons for discontinuation (i.e. inefficacy, side effects, or adverse events), infections, concomitant GCs, csDMARDs, and NSAIDs were also recorded in our cohort of patients.

### Treatment response

Treatment response was evaluated at 6 and 12 months after the first administration, and every year thereafter until 48 months.

#### Effectiveness outcomes

Relevant patient-reported outcomes (PROs) [36], such as the Visual Analogue Scale of pain (VAS-pain), global health (VAS-GH), and Physician (VAS-PH), Health Assessment Questionnaires modified for spondyloarthritis (HAQ-S), Bath Ankylosing Spondylitis Functional Index (BASFI), and Bath Ankylosing Spondylitis disease activity index (BASDAI) were collected in all participating patients. The clinical evaluation, performed by an experienced rheumatologist and an experienced dermatologist (the same assessor at each time point), included the Psoriasis Area and Severity Index (PASI), the assessment of the presence of psoriatic onychopathy and dactylitis (yes/no), joint tenderness (in 68 joints) and swollen joint count (in 66 joints), as well as the Disease Activity in Psoriatic Arthritis (DAPSA) score and the Ankylosing Spondylitis Disease Activity Score (ASDAS) based on C-reactive protein (CRP) [36]. Enthesitis was assessed using the Leeds Enthesitis Index (LEI), and dactylitis was expressed as the number of digits involved. Biochemical acute phase reactants (erythrocyte sedimentation rate = ESR and CRP) value were measured and analyzed. Our laboratory’s reference ranges were as follows: ESR 0–25 mm/h; CRP 0–6 mg/L.

#### Composite measures of disease activity

The percentage of PsA patients achieving low disease activity was assessed by minimal disease activity (MDA), a Coates’ composite measure that requires the fulfillment of five out of the seven criteria, [37] and by DAPSA disease activity response able to classify patients into remission (REM), low (LDA), moderate (MoDA) or high (HDA) disease activity (with cut-off values of ≤ 4 for REM, > 4 and ≤ 14 for LDA, > 14 and ≤ 28 for MoDA, and > 28 for HDA) [38]. MDA and DAPSA disease activity states were also calculated after subdividing the PsA population into two subgroups, according to the line of bDMARDs (naïve vs. non-naïve patients), and in 3 subgroups according to the presence and number of comorbidities (no comorbidities vs. 1–3 comorbidities vs. > 3 comorbidities).

### Treatment retention

The overall retention of secukinumab was defined as the probability of long-term drug survival of up to 4 years of treatment, as shown by Kaplan–Meier curves. The drug retention rate (DRR) was calculated as the number of days the patient remained on therapy. The treatment initiation date was the day the first dose was administered and the stop date was the day the treatment was definitively discontinued.

### Statistical analysis

A descriptive analysis of the collected data was performed. Data were expressed as frequencies and percentages for categorical variables, and as median and interquartile range (IQR) for continuous variables. Patients’ characteristics were compared between naïve and non-naïve using the Chi-square test or Fisher’s exact test for categorical variables, and the t-test or the Wilcoxon rank test or ANOVA (Kruskal Wallis) for continuous variables, based on data distribution. Effectiveness measures and outcome data were compared between baseline and 48-months using the Chi-square test or the Wilcoxon rank test, as appropriate. Kaplan–Meier curves were used to assess the cumulative DRR of secukinumab with the event being drug discontinuation due to inefficacy/adverse event. Furthermore, Kaplan–Meier curves were also employed to evaluate the impact of comorbidities, patient clinical characteristics, and concomitant medications on the DRR of secukinumab. Survival curves were compared using the log-rank test. All statistical analyses were carried out with the SPSS 13.0 software (SPSS Inc., IL, USA). Two-tailed p-values lower than 0.05 were considered statistically significant.

## Results

### Patients’ characteristics

In total, 685 PsA patients were enrolled [42.5% male; median age 57 years (49–64)] with median disease duration of 9 years, and median treatment duration of 36 (16–55) months. Secukinumab was the first-line biologic treatment in 225 patients (32.9%) (naïve), and the second-(or more) line biologic treatment in 460 patients (67.2%) (non-naïve); 444 patients (64.8%) received monotherapy. At baseline, 339 (49.5%) patients were receiving secukinumab 150 mg/injection and 346 (50.5%) patients secukinumab 300 mg/injection. The patients’ clinical and laboratory baseline (T0) characteristics, such as concomitant treatments, are summarized in Table 1.

**Table 1:** Baseline characteristics of 685 PsA patients treated with secukinumab over a 48-month follow-up period

PsA Features	Total patients	NAÏVE vs	NON-NAÏVE	*p*
**Male sex, n (%)**	291 (42.5)	96 (42.7)	195 (42.4)	*ns*
**Female sex, n (%)**	394 (57.5)	129 (57.3)	265 (57.6)	*ns*
**Age (years), median (IQR)**	57 (49–64)	55 (48–62)	57 (49–64)	*0.05*
**Age of diagnosis (years), median (IQR)**	48 (37–54)	48 (39.5–55)	46 (36–53)	*ns*
**Age of disease onset (years), median (IQR)**	45 (31–56)	45 (32.5–55)	42.5 (30–57)	*0.04*
**Disease duration (years), median (IQR)**	9 (6–14.3)	6 (4–10)	11 (7–17)	*0.001*
**PsA, n (%)**	**685**	**225**	**460**	*N/A*
*Polyarticular*	354 (51.7)	100 (44.4)	254 (55.2)	*0.03*
*Mono/Oligoarticular*	195 (28.5)	81 (36)	114 (24.8)	*0.04*
*Axial involvement*	219 (31.9)	75 (33.3)	144 (31.3)	*ns*
*Only axial involvement*	111 (16.2)	40 (17.8)	71 (15.4)	*ns*
*Axial and peripheral involvement*	108 (15.8)	35 (15.6)	73 (15.9)	*ns*
*Only DIP joint involvement*	22 (3.2)	4 (1.8)	18 (3.9)	*ns*
*Arthritis mutilans*	3 (0.4)	0 (0)	3 (0.7)	*ns*
*Enthesitis*	330 (48.2)	107 (47.6)	223 (48.5)	*ns*
*Dactylitis*	115 (16.8)	39 (17.3)	76 (16.5)	*ns*
**Age of psoriasis onset (years), median (IQR)**	39 (24.8–48)	39.5 (25–49.8)	40 (25–50)	*ns*
**Psoriasis, n (%)**	430 (62.8)	134 (59.6)	296 (64.3)	*0.04*
**Onychopathy, n (%)**	249 (36.4)	88 (39.1)	161 (35.0)	*0.04*
**IBD, n (%)**	29 (4.2)	13 (5.8)	16 (3.5)	*ns*
**Uveitis, n (%)**	20 (2.9)	6 (2.7)	14 (3.1)	*ns*
**Family history of psoriasis or SpA, N (%)**	247 (36.1)	81 (36.0)	166 (36.1)	*ns*
**Erosions, n (%)**	127 (18.5)	41 (18.2)	86 (18.7)	*ns*
**Weight (kg), median (IQR)**	74 (64–84.5)	71 (60–84)	75 (65–85)	*ns*
**Height (cm), median (IQR)**	167.5 (160–175)	167 (160–175)	168 (160–175)	*ns*
**BMI, median (IQR)**	25.7 (23.4–28.9)	24.78 (22.7–28.6)	26.2 (23.6–29.1)	*ns*
**TJ [0–68]** **, median (IQR)**	6 (2–11)	6 (2–11)	6 (2–11)	*ns*
**SJ [0–66], median (IQR)**	1 (0–3)	1 (0–2)	3 (1–3)	*0.04*
**LEI [0–6], median (IQR)**	2 (0–3)	2 (0–3)	2 (0–3)	*ns*
**Dactylitis [0–20] number of digits, median (IQR)**	0.7 (0.4–1.9)	0.7 (0.4–1.5)	0.9 (0.8–1.3)	*ns*
**PASI [0–72], median (IQR)**	3.2 (1.2–5.6)	3.1 (1.3–4.4)	4.1 (2.1–5.5)	*0.01*
**ESR [0–25] (mm/h), median (IQR)**	15 (7–27)	15 (7–24.3)	15 (7–28)	*ns*
**CRP [0–6] (mg/L), median (IQR)**	3.3 (1.3–7.9)	3.2 (1.3–7.9)	3.6 (1.2–7.9)	*ns*
**DAPSA [0–164], median (IQR)**	23.6 (17.1–30.5)	23 (17.1–30)	23 (17–30.8)	*ns*
**ASDAS-CRP [0–6], median (IQR)**	3.1 (2.3–3.6)	3.1 (2.5–3.5)	3.3 (2.8–3.8)	*0.05*
**HAQ-S [0–8], median (IQR)**	1.3 (0.9–1.8)	1 (0.8–1.7)	1.4 (1.0–1.9)	*0.05*
**VAS-pain [0–10], median (IQR)**	7 (6–8)	7 (6–8)	7 (6–8)	*ns*
**VAS-GH [0–10], median (IQR)**	7 (5–8)	7 (5–7)	7 (5–8)	*ns*
**VAS-PH [0–10], median (IQR)**	7 (5–7)	7 (5–7)	7 (5–7)	*ns*
**BASDAI [0–10], median (IQR)**	5.5 (4.2–6.9)	5.5 (4.7–6.7)	5.5 (4.8–7.2)	*ns*
**BASFI [0–10], median (IQR)**	6 (4.4–7)	6 (4.8–7)	5.8 (4.1–7.0)	*ns*
**Treatment duration (months), median (IQR)**	42 (16–55)	42 (16–55.5)	43 (17–56.5)	*ns*
**Dosage 300 mg/injection, n (%)**	339 (49.5)	39 (17.3)	300 (65.2)	< *0.01*
**Dosage 150 mg/injection, n (%)**	346 (50.5)	186 (82.7)	160 (34.8)	< *0.01*
**1st line, n (%)**	225 (32.8)	225 (100)	0 (0)	*N/A*
**Failure biologic drugs, n (%)**	460 (67.2)	0 (0)	460 (100)	*N/A*
**2nd line, n (%)**	179 (26.1)	0 (0)	179 (38.9)	*N/A*
**3rd line, n (%)**	141 (20.6)	0 (0)	141 (30.7)	*N/A*
**4th line, n (%)**	84 (12.3)	0 (0)	84 (18.3)	*N/A*
**5th line or more, n (%)**	56 (8.2)	0 (0)	56 (12.2)	*N/A*
**Concomitant NSAIDs, n (%)**	346 (50.5)	98 (43.6)	248 (53.9)	*0.04*
**Concomitant steroid, n (%)**	146 (21.3)	31 (13.8)	115 (25.0)	*0.03*
**Concomitant csDMARDs, n (%)**	241 (35.2)	83 (36.9)	159 (34.6)	*ns*

*Legend*: Data are expressed as median (interquartile range [IQR]) unless otherwise specified; range of possible values are indicated in square brackets. p§ ANOVA (Kruskal Wallis) at T0: *p* < 0.05

*SpA* spondyloarthritis, *PsA* psoriatic arthritis, *naïve* naïve to bDMARDs, *non-naïve* bDMARDs failure, *DIP* distal interphalangeal, *IBD* inflammatory bowel disease, *PASI* Psoriasis Area Severity Index, *TJ* Tender Joint, *SJ* Swollen Joint, *LEI* Leeds Enthesitis Index, *DAPSA* Disease Activity Index for Psoriatic Arthritis, *kg* kilogram, *cm* centimeter, *BMI* body mass index, *ESR* erythrocyte sedimentation rate, *CRP* C-reactive protein, *VAS-pain* Visual Analogue Scale-pain, *VAS-GH* Visual Analogue Scale-global health, *BASDAI* Bath Ankylosing Spondylitis Disease Activity Index, *BASFI* Bath Ankylosing Spondylitis Functional Index, *ASDAS* Ankylosing Spondylitis disease activity score, *HAQ-S* Health Assessment Questionnaire modified for spondyloarthritis, *NSAIDs* non-steroidal anti-inflammatory drugs, *csDMARDs* conventional synthetic disease-modifying antirheumatic drugs, *ns* not statistically significant, *N/A* not applicable

Polyarthritis was a prominent manifestation in 51.7% of cases; asymmetric oligoarthritis or monoarthritis in 28.5%; axial involvement with sacroiliitis and/or spondylitis in 31.9%; and enthesitis in 48.2% of patients. Erosive disease was recorded at baseline in 127 patients (18.5%), arthritis mutilans in 3 cases (0.4%), and prominent distal interphalangeal joint (DIP) involvement in 22 cases (3.2%). The following extra-articular manifestations were recorded: inflammatory bowel disease (IBD) (4.2%, *n* = 29) and uveitis (2.9%, *n* = 20) in remission.

At T0, 241 patients (35.2%) were receiving concomitant csDMARDs, 346 (50.5%) were on NSAIDs, and 146 (21.3%) were taking GCs.

At T0, non-naïve (as compared to naïve) had: a more polyarticular pattern with higher frequency of swollen joints; a longer disease and psoriasis duration; a greater prevalence of psoriasis and onychopathy; a higher GCs intake; and worse functional and disease activity indices. No significant differences were found as it pertains to enthesitis, dactylitis and extra-articular features, and the other clinical and functional parameters (Table 1).

### Therapy effectiveness

Of the 685 PsA patients, 608 (88.8%; naïve *n* = 207; non-naïve *n* = 401) were evaluated at T6, 526 (76.8%; naïve *n* = 176; non-naïve *n* = 350) at T12, 390 (56.9%; naïve *n* = 146; non-naïve *n* = 244) at T24, 315 (45.9%; naïve *n* = 126; non-naïve *n* = 189) at T36 and 240 (35.0%; naïve *n* = 104; non-naïve *n* = 136) at T48.

The whole population achieved a significant decrease in tender/swollen joints (T/SJ), dactylitis count, VAS-pain, VAS-GH and VAS-PH scores, PASI, LEI, HAQ-S, BASDAI, BASFI, and CRP (Supplementary Table 1). A significant improvement in ASDAS-CRP [T0 = 3.1 (2.3–3.6) vs. T48 = 1.2 (0.6–2.0); *p* = 0.02] and DAPSA [T0 = 23.6 (17.1–30.5) vs. T48 = 4.2 (2.0–10.0); *p* < 0.01] was also observed.

During the 48-month follow-up, a significantly reduced number of patients were observed with: active tender (TJC) and swollen joint count (SJC) [TJC T0 = 90.4% (*n* = 615) vs. T48 = 42.5% (*n* = 102); SJC T0 = 61.2% (*n* = 419) vs. T48 = 13.3% (*n* = 32); *p* < 0.01]; enthesitis T0 = 48.2% (*n* = 330) vs. T48 = 10.8% (*n* = 26); *p* < 0.01; dactylitis T0 = 16.8% (*n* = 115) vs. T48 = 5.8% (*n* = 14); *p* < 0.01; and psoriasis T0 = 62.8% (*n* = 430) vs. T48 = 10.0% (*n* = 24); *p* < 0.01.

Overall, at T48, secukinumab appeared to be effective in both naïve and non-naïve patients, albeit with a lower reduction in disease activity in the latter. Naïve patients showed better physical function and lower inflammatory activity vs. non-naïve patients [VAS-GH 1 (0–4) vs. 3 (1–5) (*p* = 0.04); ESR 8.0 (5.0–12.7) vs. 10 (15–16.8) (*p* = 0.04); BASFI 0.8 (0.1–2.0) vs. 1.3 (0.0–2.7) (*p* = 0.04); BASDAI 0.3 (0.0–2.1) vs. 1.6 (0.0–3.0) (*p* = 0.04)]. However, non-naïve patients maintained higher disease activity indices than naïve [DAPSA 4.0 (1.4–8.1) vs. 6.0 (2.2–10.4) (*p* = 0.04); ASDAS-CRP 1.1 (0.3–1.6) vs. 1.72 (0.76) (*p* = 0.05)] (Table 2).

**Table 2:** Clinical, functional, disease activity, and serological parameters of naïve (*n* = 225) and biologic agents failure (*n* = 460) PsA patients over a 48-month follow-up period

	**T0**	**T6**	**T12**	**T24**	**T36**	**T48**
**TJ [0–68], median (IQR)**						
Naïve	6 (2–11)	4 (0–5)	4 (0–1)	2 (0–1)	1 (0–1)	1 (0–1)
Non-naïve	6 (2–11)	4 (0–5)	2 (0–4)	2 (0–1)	1 (0–2)	1 (0–2)
*p*	*ns*	*ns*	*ns*	*ns*	*ns*	*ns*
**SJ [0–66], median (IQR)**						
Naïve	1 (0–2)	1 (0–1)	0.5 (0–1)	0.0 (0–0.5)	0.0 (0.0–0.2)	0.0 (0.0–0.0)
Non-naïve	3 (1–3)	1 (0–1)	0.7 (0–1.2)	0.2 (0–0.8)	0.1 (0.0–0.6)	0.0 (0.0–0.1)
*p*	*p* = *0.05*	*ns*	*ns*	*ns*	*ns*	*ns*
**LEI [0–6], median (IQR)**						
Naïve	2 (0–3)	0.1 (0–2)	0.1 (0–1)	0.0 (0–0.3)	0.0 (0–0.1)	0.0 (0.0–0.0)
Non-naïve	2 (0–3)	0.4 (0–2)	0.3 (0–1)	0.2 (0–0.7)	0.1 (0–0.8)	0.0 (0.0–0.2)
*p*	*ns*	*ns*	*ns*	*ns*	*ns*	*ns*
**Dactylitis [0–20], median (IQR)**						
Naïve	0.7 (0.4–0.9)	0.5 (0.2–0.7)	0.2 (0.1–0.6)	0.1 (0.0–0.5)	0.0 (0.0–0.1)	0.0 (0.0–0.0)
Non-naïve	0.9 (0.8–1.3)	0.6 (0.2–0.9)	0.3 (0.1–0.9)	0.4 (0.2–0.9)	0.0 (0.0–0.2)	0.0 (0.0–0.2)
*p*	*ns*	*ns*	*ns*	*ns*	*ns*	*ns*
**PASI [0–72], median (IQR)**						
Naïve	3.1 (1.3–4.4)	0.4 (0.1–3.3)	0.1 (0.0–2.1)	0.1 (0.0–1.7)	0.0 (0.0–1.0)	0.0 (0.0–0.5)
Non-naïve	4.1 (2.1–5.5)	0.8 (0.2–4.1)	0.4 (0.0–2.5)	0.6 (0.0–2.1)	0.2 (0.0–1.3)	0.1 (0.0–0.8)
*p*	*ns*	*ns*	*ns*	*ns*	*ns*	*ns*
**ESR [0–25] (mm/h), median (IQR)**						
Naïve	15 (7–24.3)	10 (5–20)	9 (4–16)	9 (5.0–12.5)	9 (5.0–15.0)	8 (5.0–12.7)
Non-naïve	15 (7–28)	12 (6–24)	12 (6–20)	12 (5–20)	12 (6–18)	10 (15–16.8)
*p*	*ns*	*ns*	*p* = *0.05*	*p* = *0.05*	*p* = *0.05*	*p* = *0.05*
**CRP [0–6] (mg/L), median (IQR)**						
Naïve	3.2 (1.3–7.9)	2.2 (1.0–4.0)	2.3 (1.0–4.0)	2.0 (1.0–3.2)	2.0 (1.0–3.0)	2.0 (1.0–3.3)
Non-naïve	3.6 (1.2–7.9)	2.9 (1.2–6.0)	2.1 (1.0–5.0)	2.0 (1.0–4.0)	2.0 (1.0–3.7)	2.0 (1.0–3.8)
*p*	*ns*	*ns*	*ns*	*ns*	*ns*	*ns*
**DAPSA [0–164], median (IQR)**						
Naïve	23 (17.1–30)	11.1 (6.4–17.9)	9 (4.5–14.8)	5.2 (1.3–11.8)	4.7 (2.1–10.3)	4.0 (1.4–8.1)
Non-naïve	23 (17–30.8)	13.5 (8.9–20.5)	10 (6.0–17.4)	8.1 (3.3–14.1)	6 (2.0–12.3)	6.0 (2.2–10.4)
*p*	*ns*	*p* = *0.05*	*p* = *0.05*	*p* = *0.04*	*p* = * 0.05*	*p* = *0.04*
**ASDAS-CRP [0–6], median (IQR)**						
Naïve	3.1 (2.5–3.5)	2.1 (1.4–2.6)	1.5 (1.0–2.5)	1.2 (0.7–2.0)	1.2 (0.6–1.7)	1.1 (0.3–1.6)
Non-naïve	3.3 (2.8–3.8)	2.2 (1.3–3.1)	2.0 (1.2–2.7)	1.5 (1.0–2.2)	1.5 (0.8–2.2)	1.72 (0.76)
*p*	*ns*	*ns*	*p* = *0.05*	*ns*	*ns*	*p* = *0.05*
**HAQ-S [0–8], median (IQR)**						
Naïve	1 (0.8–1.7)	0.75 (0.2–1.0)	0.5 (0.0–1.0)	0.1 (0.0–0.5)	0.1 (0.0–0.5)	0.0 (0.0–0.4)
Non-naïve	1.4 (1.0–1.9)	1.0 (0.5–1.3)	0.8 (0.3–1.2)	0.5 (0.1–1.2)	0.4 (0.0–0.8)	0.3 (0.0–0.7)
*p*	*ns*	*ns*	*ns*	*ns*	*ns*	*ns*
**VAS-pain [0–10], median (IQR)**						
Naïve	7 (6–8)	4 (2–6)	3.5 (2–5)	2.5 (1–4)	2 (0–4)	1 (0–4)
Non-naïve	7 (6–8)	5 (3–7)	4 (2–6)	3 (1–5)	2 (1–5)	2 (1–4)
*p*	*ns*	*ns*	*ns*	*ns*	*ns*	*ns*
**VAS-GH [0–10], median (IQR)**						
Naïve	7 (5–7)	4 (3–6)	4 (2–6)	2 (1–5)	2 (0–4)	1 (0–4)
Non-naïve	7 (5–8)	5 (3–6)	4 (3–6)	4 (2–6)	3 (1–6)	3 (1–5)
*p*	*ns*	*ns*	*ns*	*p* = *0.04*	*p* = *0.05*	*p* = *0.04*
**VAS-PH [0–10], median (IQR)**						
Naïve	7 (5–7)	4 (2–5)	4 (1–2)	2 (0–3)	1 (0–3)	1 (0–3)
Non-naïve	7 (5–7)	4 (3–6)	3 (1–5)	2 (1–5)	1 (0.0–4.5)	1 (0–3)
*p*	*ns*	*ns*	*ns*	*ns*	*ns*	*ns*
**BASDAI [0–10], median (IQR)**						
Naïve	5.5 (4.7–6.7)	3.5 (2.0–4.1)	2.2 (1.3–4.3)	1.1 (0.3–2.3)	1.0 (0.0–2.4)	0.3 (0.0–2.1)
Non-naïve	5.5 (4.8–7.2)	3.6 (2.0–5.4)	2.9 (1.8–4.8)	2.1 (0.8–4.1)	1.9 (0.2–3.9)	1.6 (0.0–3.0)
*p*	*ns*	*ns*	*ns*	*p* = *0.05*	*p* = *0.05*	*p* = *0.04*
**BASFI [0–10], median (IQR)**						
Naïve	6 (4.8–7)	3.3 (2.3–4.6)	2.0 (1.5–3.0)	1.0 (0.8–2.3)	1.0 (0.2–2.0)	0.8 (0.1–2.0)
Non-naïve	5.8 (4.1–7.0)	4.1 (2.5–5.5)	3.0 (1.9–4.5)	2.1 (1.1–3.5)	1.7 (0.8–2.2)	1.3 (0.0–2.7)
*p*	*ns*	*ns*	*ns*	*p* = *0.05*	*p* = *0.05*	*p* = *0.04*

Data are expressed as median (interquartile range [IQR]). *p* ≤ 0.05. Values were computed by chi-square test (for proportion) or Wilcoxon test (for continuous data)

*Legend*: *Naïve* naïve to bDMARDs, *Non-naïve* bDMARDs failure, *TJ* Tender Joint, *SJ* Swollen Joint, *LEI* Leeds Enthesitis Index, *PASI* Psoriasis Area Severity Index, *ESR* erythrocyte sedimentation rate, *CRP* C-reactive protein, *DAPSA* Disease Activity Index for Psoriatic Arthritis, *ASDAS* Ankylosing Spondylitis Disease Activity Score, *HAQ-S* Health Assessment Questionnaire modified for spondyloarthritis, *VAS-pain* Visual Analogue Scale-pain, *VAS-GH* Visual Analogue Scale-global health, *BASDAI* Bath Ankylosing Spondylitis Disease Activity Index, *BASFI* Bath Ankylosing Spondylitis Functional Index, *ns* not statistically significant

**Fig. 1:**
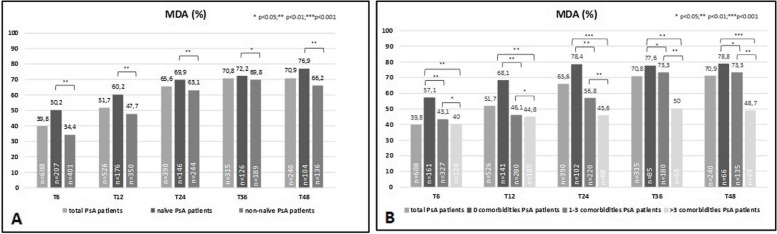
**A-B** Minimal disease activity (MDA) (percentage, %) of overall population and after their subdivision in two groups, according to the DMARD treatment line (naïve patients vs non-naïve patients) (**A**) and after their subdivision in three groups, according to number of comorbidities (0 comorbidities, 1–3 comorbidities, > 3 comorbidities) (**B**). Legend: n, number of evaluated patients; naïve: naïve to bDMARDs; non-naïve: bDMARDs failure

**Fig. 2:**
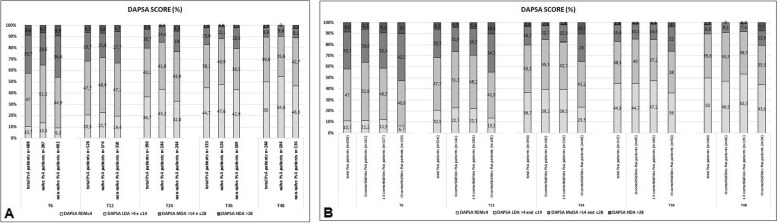
**A-B** Disease Activity for Psoriatic Arthritis (DAPSA) score (percentage, %) of overall population and after their subdivision in two groups, according to the DMARD treatment line (naïve patients vs non-naïve patients) (**A**) and after their subdivision in three groups, according to number of comorbidities (0 comorbidities, 1–3 comorbidities, > 3 comorbidities) (**B**). Legend: n, number of evaluated patients; naïve: naïve to bDMARDs; non-naïve: bDMARDs failure

During follow-up, a higher proportion of the study population achieved MDA and improvement in DAPSA: MDA 65.6% and 70.9% at T24 and T48, respectively (Fig. 1A). Similarly, 36.7%/43.1% achieved a DAPSA-REM/DAPSA-LDA score at T24, and 50%/39.6% achieved a DAPSA-REM/DAPSA-LDA score at T48 (Fig. 2A). At T48, 76.9% of naïve and 66.2% of non-naïve patients achieved MDA (*p* < 0.01) (Fig. 1A). The number of patients who achieved MDA according to the presence and number of comorbidities was also ascertained. More patients without comorbidities achieved MDA than those with comorbidities (Fig. 1B), and there was an inverse correlation with the number of comorbidities [%MDA no comorbidities vs. 1–3 comorbidities = 78.8% vs. 73.3% (*p* < 0.05); no comorbidities vs. > 3 comorbidities = 78.8% vs. 48.7% (*p* < 0.001). Overall, the rates of DAPSA-REM and DAPSA-LDA were higher in naïve patients than non-naïve patients (Fig. 2A), although both indices were similar among those without comorbidities and those with 1–3 comorbidities, and lower in those with more than 3 comorbidities (Fig. 2B).

The proportion of patients receiving csDMARDs decreased steadily from T0 [35.2%, *n* = 241] to T6 [34.4%, *n* = 209], T12 [32.3%, *n* = 170], T24 [36.7%, *n* = 143], T36 [22.2%, *n* = 70], and T48 [23.3%, *n* = 56]. Similarly, patients treated with GCs decreased from T0 [21.3%, *n* = 146], 12.7% (*n* = 77) at T6, 10.1% (*n* = 53) at T12, 7.7% (*n* = 30) at T24, 8.3% (*n* = 26) at T36 and 8.8% (*n* = 21) at T48. A marked reduction in NSAIDs intake was also observed from T0 [50.5%, *n* = 346] to T6 [38.7%, *n* = 235], T12 [32.5%, *n* = 171], T24 [31.0%, *n* = 121], T36 [29.8%, *n* = 94] and T48 [30.4%, *n* = 73]. Throughout the follow-up, a greater reduction in patients taking csDMARDs and GCs was found in non-naïve patients versus naïve patients [29.4%, *n* = 40 vs. 15.4%, *n* = 16; and 12.5%, *n* = 17 vs. 3.8%, *n* = 4 at T48, respectively], whereas the percentage of patients taking NSAIDs decreased comparably between naïve vs. non-naïve patients [31.7%, *n* = 33 vs. 29.4%, *n* = 40 at T48].

### Drug retention rate

**Fig. 3:**
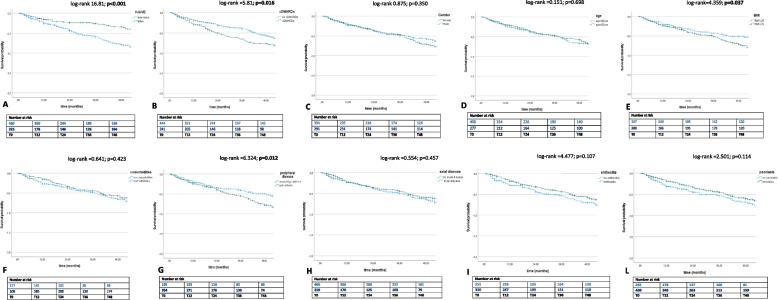
**A-L** Drug survival in the overall population and after their subdivision in two groups, according to the DMARD treatment line (naïve vs non-naïve patients) (**A**), use of combined csDMARDs (no csDMARDs vs. csDMARDs) (**B**), gender (male vs. female) (**C**), age (< 60 years patients vs < 60 years patients) (**D**), BMI (overweight vs. normal weight) (**E**), comorbidities (patients without comorbidities vs patients with comorbidities (**F**), disease phenotype (mono/oligoarthritis vs. polyarthritis (**G**), no axial disease vs. axial disease (**H**), no enthesitis vs. enthesitis (**I**), and no psoriasis vs. psoriasis (**L**)

The DRR at T48 was good (66%) in the whole study population (Fig. 3 A-L), with some differences according to the choice of bDMARDs treatment (naïve vs. non-naïve; log-rank = 16.81; *p* < 0.001), the use of combination therapy with csDMARDs (no csDMARDs vs. csDMARDs; log-rank = 5.81; *p* = 0.016), the type of peripheral disease (mono/oligoarthritis vs. polyarthritis; log-rank = 6.324; *p* = 0.012) and the BMI (subjects with BMI ≤ 25 vs. BMI > 25; log-rank = 4.359; *p* = 0.037). The Kaplan–Meier curves did not show any differences between patients < 60 years old vs. patients > 60 years old (log-rank = 0.151; *p* = 0.698), subjects without vs. with comorbidities (log-rank = 0.641; *p* = 0.423), male vs. female (log-rank = 0.875; *p* = 0.350), subjects without vs. with axial involvement (log-rank = 0.554; *p* = 0.457), patients without vs. with enthesitis (log-rank = 4.477; *p* = 0.107), and patients without vs. with psoriasis (log-rank = 2.501; *p* = 0.114).

### Comorbidities

Among the PsA population, 177 patients (25.8%) had no comorbidities, 371 patients (54.2%) had 1–3 comorbidities and 137 patients (20%) had > 3 comorbidities.

The most common comorbidities were: hypertension (35.8%, *n* = 245), dyslipidemia (27.9%, *n* = 191), fibromyalgia (19.1%, *n* = 131), thyroid disorders (12.3%, *n* = 84), metabolic syndrome (MetS) (10.9%, *n* = 75), liver disease (e.g. steatosis) (10.9%, *n* = 75), hyperuricemia (10.7%, *n* = 73), osteoporosis (9.9%, *n* = 68), type II diabetes (9.8%, *n* = 67), depression (9.6%, *n* = 66), gastritis, gastric ulcer or dyspeptic disorders (8.9%, *n* = 61), positive Mantoux TB skin test or QuantiFERON-TB Gold test (7.9%, *n* = 54) without active tuberculosis, ischemic heart disease (7.7%, *n* = 53), previously eradicated cancer (6.4%, *n* = 44), pneumopathies (6.1%, *n* = 42), neurological disorders (e.g. neuropathy) (5.8%, *n* = 40), previous hepatitis B (4.8%, *n* = 33), previous hepatitis C (2.2%, *n* = 15), kidney failure (2.1%, *n* = 14). The frequency of these comorbidities were described in both naïve and non-naïve patients (Supplementary Table 2). A higher prevalence of cardiovascular disorders, diabetes, hepatic steatosis and MetS was reported in non-naïve patients.

### Safety and discontinuation

#### Reasons for discontinuation

Secukinumab was found to be safe and well tolerated (Table 3).

**Table 3:** Reasons for discontinuation in naïve (*n* = 225) and biologic agents failure (*n* = 460) PsA patients

	**Total patients**	**Naïve**	**Non-naïve**	***p§***
**Reasons for discontinuation**	**233 (34.1%)**	**51 (22.7%)**	**182 (39.6%)**	***0.04***
***Primary loss of efficacy***	**61 (8.9%)**	**15 (6.7%)**	**46 (10.0%)**	***0.03***
***Secondary loss of efficacy***	**109 (15.9%)**	**18 (8.0%)**	**91 (19.8%)**	***0.03***
***Adverse events (AE)***	**41 (5.9%)**	**11 (4.9%)**	**30 (6.5%)**	***ns***
*Reactions at the injection site or skin manifestations*	11 (1.6%)	4 (1.8%)	7 (1.5%)	*ns*
*Leuko/neutropenia*	1 (0.1%)	0 (0%)	1 (0.2%)	*ns*
*Dyspnea*	2 (0.3%)	0 (0%)	2 (0.4%)	*ns*
*Hypertransaminasemia*	1 (0.1%)	0 (0%)	1 (0.2%)	*ns*
*Gastrointestinal disorders (nausea, diarrhea, abdominal pain)*	6 (0.9%)	2 (0.9%)	4 (0.9%)	*ns*
*IBD flare-up*	1 (0.1%)	0 (0%)	1 (0.2%)	*ns*
*Severe recurrent infections*	11 (1.6%)	3 (1.3%)	8 (1.7%)	*ns*
*Multiple sclerosis*	1 (0.1%)	0 (0%)	1 (0.2%)	*ns*
*Onset of new cancer*	7 (1.0%)	2 (0.9%)	5 (1.1%)	*ns*
***Other reasons for drop-out***	**22 (3.2%)**	**7 (3.1%)**	**15 (3.3%)**	***ns***
*Pregnancy*	3 (0.4%)	1 (0.4%)	2 (0.4%)	*ns*
*Non-compliance*	2 (0.3%)	1 (0.4%)	1 (0.2%)	*ns*
*Remission*	3 (0.4%)	1 (0.4%)	2 (0.4%)	*ns*
*Lost to follow-up*	14 (2.0%)	4 (1.8%)	10 (2.2%)	*ns*
**Infectious events**	**146 (21.3%)**	**41 (18.2%)**	**105 (22.8%)**	***0.04***
*COVID-19 infection*	27 (3.9%)	6 (2.7%)	21 (4.6%)	*0.05*
*Other non-COVID-19 respiratory tract infections*	37 (5.4%)	11 (4.9%)	26 (5.7%)	*ns*
*Oral or vaginal candidiasis*	21 (3.1%)	4 (1.8%)	17 (3.7%)	*0.04*
*Herpetic labial infections*	13 (1.9%)	5 (2.2%)	8 (1.7%)	*ns*
*Herpes zoster*	6 (0.9%)	1 (0.4%)	5 (1.1%)	*0.04*
*Gastroenteritis or diverticulitis*	11 (1.6%)	3 (1.3%)	8 (1.7%)	*ns*
*Urinary tract infections*	29 (4.2%)	10 (4.4%)	19 (4.1%)	*ns*
*Septic arthritis*	2 (0.3%)	1 (0.4%)	1 (0.2%)	ns

*Legend*: Values are expressed as frequencies (%) unless otherwise specified. p§ ANOVA (Kruskal Wallis): *p* < 0.05 naïve vs. non-naïve

*Naïve* naïve to bDMARDs, *non-naïve* bDMARDs failure, *AE* adverse event, *IBD* inflammatory bowel disease, *COVID-19* Coronavirus disease 2019, *ns* not statistically significant

In total, 233 patients (34.1%) discontinued treatment during the follow-up, mainly due to primary and secondary loss of effectiveness (61 and 109, respectively). Another 22 patients dropped out of the observational study. Ultimately, only 41 patients discontinued secukinumab due to adverse events (mainly for skin manifestations in the injection site (11) and severe recurrent infections (11), followed by gastrointestinal disorders (6); only 1 patient had an IBD flare-up and 7 patients an onset of new cancer diagnosis). Liver and kidney functions were monitored in all patients throughout the study period, and only 2 patients exhibited abnormal values.

#### Infections

Overall, 146 episodes of mild infection were recorded over the study period, all resolved following oral antimicrobial therapy, without hospitalization or drug discontinuation. Eleven patients developed severe infections, prompting a discontinuation of SEC (5 bronchopneumonia, 2 urosepsis due to Escherichia coli, 1 erysipelas with sepsis due to Staphylococcus aureus with hospitalization, and 3 recurrent candidiasis). There were no differences between naïve and non-naïve patients as regards safety — except for a slightly increased frequency of herpes infections and candidiasis in non-naïve patients (Table 3).

## Discussion

This study provided an assessment of the effectiveness, safety, and drug retention rate of secukinumab in patients with PsA in a real-life multicenter cohort followed up for 4 years. To the best of our knowledge, to date no other studies have been conducted for such a long observation period and in such a large cohort of patients. Furthermore, this study focused on the impact of previous biologic treatment failure, clinical disease features, and comorbidities on achievement of good clinical control of disease activity and on drug survival.

Our previous prospective multicenter study in 608 patients with moderate-to-severe PsA, published elsewhere, found that secukinumab was safe and effective in PsA, as shown by a significant decrease in all clinical, functional, and disease activity outcomes over a 24-month follow-up period [22]. These findings confirmed its notable effectiveness on all PsA domains (arthritis, enthesitis, dactylitis, spine symptoms such as those on psoriasis, and PROs and inflammatory markers), regardless of the biologic treatment line. In this study conducted on 685 PsA patients followed up for 48 months, we found that this improvement was maintained or even increased after 3 and 4 years of treatment, and was numerically better — albeit not significantly — in biologic-naïve patients, indicating that secukinumab may be effective in both naïve and non-naïve patients, in line with previous reports in the literature [27, 28, 39, 40]. Our findings further confirmed that secukinumab was effective across all GRAPPA-OMERACT PsA core domains. These results are similar to those reported in the pooled analysis of 2049 patients in the FUTURE 2–5 trials, which suggested that PsA can benefit from secukinumab across the whole clinical phenotype spectrum commonly encountered in this disease [41]. Furthermore, post hoc analysis of FUTURE 2 data showed that secukinumab treatment resulted in Psoriatic Arthritis Disease Activity Score (PASDAS) remission or LDA at week 16 with responses sustained or further improved through week 104 [42]. Furthermore, in our study MDA was achieved in 65.6% at T24, and this proportion increased to 70.9% at T48; similarly, 36.7% and 43.1% of patients achieved DAPSA-REM and DAPSA-LDA at T24, respectively, and this state was maintained or improved at T48 (DAPSA-REM in 50% and DAPSA-LDA in 39.6%). Higher MDA and DAPSA response rates were also observed in the naïve vs. non-naïve group, in line with RCT data [12, 14, 18] and real-life experiences [22, 24, 25, 31–33, 43].

Our findings indicate that secukinumab may be slightly more effective in naïve vs. non-naïve patients, as reflected by the lower reduction in disease activity indices in the latter. This corroborates previous reports in the literature that secukinumab is appropriate to use both in biologic-naïve patients and non-naïve patients with inadequate response or intolerance to TNF inhibitors (TNFi) [12, 22–25, 31–33].

Another aim of this study was to evaluate the impact of comorbidities on achieving remission or low disease activity, and persistent therapeutic effectiveness in the PsA population.

The clinical picture of PsA may be complicated by comorbidities, which could make the management of these patients more difficult [44, 45]. Regardless of the main disease, patients with comorbidities may be at higher risk of complications and mortality as well as less responsive to therapy, compared to patients with the same disease but without these conditions [29–32, 46]. A considerable number of patients [508 (74.2%) out of 685 of our PsA population] had at least 1 comorbidity. As expected, cardiometabolic diseases were the most common comorbidity in our cohort.

The most common comorbidities in our study population were hypertension and dyslipidemia. Although there appears to be a slight inverse correlation between these 2 comorbidities and drug efficacy, we were not able to confirm these trends using subanalyses due to our small sample size.

Generally speaking, comorbidities did not appear to affect clinical response to secukinumab therapy, since patients without comorbidities achieved MDA in a slightly higher proportion compared with those with comorbidities. We found similar rates of DAPSA-REM and DAPSA-LDA in patients without comorbidities and those with 1–3 comorbidities; however, patients with > 3 comorbidities had slightly lower DAPSA-REM and DAPSA-LDA. Thus, we hypothesized that secukinumab may be considered in PsA patients with comorbidities, including cardiometabolic multimorbidity. Similarly, other real-life studies found that PsA patients with hypertension, dyslipidemia, metabolic syndrome, or obesity showed sustained effectiveness and longer secukinumab drug survival, [22–25, 31–33, 47] with more improved disease activity measures and outcomes than those found in PsA patients treated with TNFi [5, 27–29, 39, 40]. In this context, IL-17A could play a central role in inflammation, endothelial dysfunction, insulin resistance, and the consequent cardiometabolic burden of patients with PsA [48].

Regarding persistence, in our PsA population, a 48-month cumulative secukinumab DRR of 66% was estimated with a median duration of 36 months of drug administration. The overall secukinumab DRR was high both in the short term and the long term, since a similar value (71%) was found at 24-months in our previous study [22]. The treatment persistence observed here is longer than that reported by a recent interim analysis of an ongoing observational study involving 1756 patients, showing a treatment retention rate of 60.5% at 3 years [24]. The DRR was also evaluated according to bDMARD lines, clinical disease pattern, and presence of comorbidities. A higher DRR in naïve patients than non-naïve patients was observed (*p* < 0.001), and this finding is consistent with previous data from phase III RCTs [12, 14] and real-world evidence studies [21–26, 31–33, 40, 43]. This study also identified some DRR differences depending on the use of combined csDMARDs (no csDMARDs vs. csDMARDs; *p* = 0.016), and type of peripheral disease (mono/oligoarthritis vs. polyarthritis; *p* = 0.012). These results may be attributable to a more severe form of PsA with polyarticular involvement requiring combination therapy. However, as previously reported in the literature by RCTs [12–14] and registry data [21–26], secukinumab could be considered a valid option for monotherapy and in non-responder patients to previous bDMARDs. Likewise, in this study overweight or obese patients affected by PsA showed a higher risk of secukinumab discontinuation. This is corroborated by several studies showing that both obesity and metabolic syndrome are associated with lower rates of response to biologic therapy and thus reducing the odds of achieving MDA, especially as it pertains to targeted immunomodulators such as TNFi [22, 45, 46, 48, 49]. Nevertheless, few studies have noted that obesity does not appear to be associated with a lower secukinumab retention rate [31, 32, 47]. In contrast, treatment persistence appeared not to be influenced by certain patient clinical characteristics, including male gender, older age, and other PsA disease characteristics. Furthermore, the presence of comorbidities did not reduce the DRR of secukinumab. Regarding the impact of clinical disease features on secukinumab response, notable effectiveness was observed in 31.9% of patients with axial involvement, measured by the significant reduction in the ASDAS-CRP and BASDAI scores. Notably, the DRR was also found to be similar between PsA patients with or without axial disease. These findings were in line with the MAXIMISE (Managing AXIal Manifestations in psorIatic arthritis with SEcukinumab) trial, which demonstrated the efficacy of secukinumab in the management of axial manifestations of PsA [50]. Consistent with our findings, Adami et al. found the secukinumab DRR to be higher in patients with prevalent axial PsA [51].

Patients with axial involvement have significantly lower levels of circulating Dkk1, a Wnt inhibitor whose levels correlate inversely with radiographic progression of PsA [52]. In addition, IL-17 appears to exert its effect on the Wnt pathway. Notably, Dkk1 was found to be underexpressed and further inhibited by IL-17 [52]. Although there is currently scarce data on Dkk1 changes after long-term IL-17i therapy, Fassio et al. reported higher serum Dkk1 and sclerostin concentrations in a cohort of patients with peripheral PsA within 6 months of secukinumab therapy [53]. Recent data on serum sclerostin concentrations after treatment with secukinumab (from the MEASURE-1 study) [54] did not reveal significant changes at weeks 52 and 104. However, these observations are difficult to interpret, as the kinetics of Wnt regulations might reveal changes in a much quicker fashion at different stages of the disease. Further studies are necessary to ascertain whether IL-17 blockade on the Wnt signaling pathway in PsA patients with peripheral and axial involvement, correlate with slower radiographic progression. Nevertheless, our results indicate that secukinumab may yield a good treatment response in PsA patients with axial involvement — as measured by disease activity indices [14–16].

Regarding safety, in terms of routine clinical practice, studies show a safety profile similar to that previously reported in RCTs and their long-term extension studies [12, 14–16, 18], but information from real-world evidence studies [21–26] is still scarce. In our study population, the drug’s safety profile was good (only 41 cases leading to drug withdrawal due to adverse events). Although this low incidence of adverse events might be linked to the fact that minor side effects may not be reported in a real-life setting, this frequency was consistent with previous real-life reports [22, 24, 25, 31–33, 43]. A pooled safety analysis from a phase III RCT supports the favorable long-term safety of secukinumab in patients with psoriasis and PsA [15]. Secukinumab has been reported to increase the incidence of upper respiratory tract infections, mucocutaneous Candida infections, and herpes simplex infections compared to placebo, however these types of infection were usually of mild-to-moderate intensity and did not lead to treatment discontinuation. In our population, 146 episodes of mild infections were recorded over the study period, all resolved following oral antimicrobial therapy, without hospitalization or drug discontinuation. Overall, the good safety profile of the drug is confirmed even in patients with concomitant infections or comorbidities [15]. Some severe cases and exacerbations of Crohn's disease have also been described [55], so caution is recommended with its use. In our study, only 1 case of IBD flare-up was reported among 29 patients overall having a history of IBD. None of our patients developed an active tuberculous disease during the course of treatment; the presence of cardiovascular or metabolic comorbidities did not limit the choice of this drug, reduce the therapeutic response, or induce early discontinuation. There were no differences between naïve and non-naïve patients as regards safety — except for a slightly increased frequency of herpes infections and candidiasis in non-naïve patients; this finding could be due to longer disease duration and a longer period of exposure to multiple drugs.

We would be remiss not to mention some of the limitations of our study. Firstly, the heterogeneous population means that our findings may not be generalizable. Secondly, the retrospective design may carry a certain risk of bias due to the lack of standardization in data collection. Thirdly, the small size of the subgroup > 3 comorbidities — which comprised 137 patients — did not allow us to draw any definitive conclusions on drug efficacy in relation to comorbidities.

Nevertheless, some of the strengths of our study are: 1) the long-term follow-up (48 months); 2) the large sample size of our study population; 3) the selection of bDMARD-naïve patients 4) the added contribution of real-life clinical practice studies to complement the results of clinical trials, providing valuable data regarding the overall safety, efficacy; and drug survival in heterogeneous patient populations, generally with comorbidities, varying clinical patterns, and previous biologic treatment failure not recorded in RCTs.

## Conclusion

This study supports the effectiveness of secukinumab, which also seems to be a valid option for multi-drug failure patients and maintained for a long observational period over 4 years; the safety of secukinumab means it can be used in patients with comorbidities, older age, higher BMI, and in particular, cardiovascular conditions and metabolic syndrome. The good response regarding clinical improvement and impact on quality of life appeared to be independent of the clinical phenotype, and therefore applicable to all PsA subtypes, including axial involvement.

## Supplementary Information

Supplementary Material 1.

## Data Availability

No datasets were generated or analysed during the current study.

## Abbreviations

PsA: Psoriatic Arthritis
csDMARDs: Conventional synthetic disease modifying anti-rheumatic drugs
PsO: Plaque psoriasis
axSpA: Axial spondyloarthritis
TNF: Tumor necrosis factor
HRQoL: Health-related quality of life
RCTs: Randomized controlled trials
DRR: Drug retention-rate
SIR: Italian Society of Rheumatology
CASPAR: ClASsification criteria for Psoriatic Arthritis
EULAR: European Alliance of Associations for Rheumatology
GRAPPA: Group for Research and Assessment of Psoriasis and Psoriatic Arthritis
BMI: Body mass index
NSAIDs: Non-steroidal anti-inflammatory drugs
GCs: Glucocorticosteroids
bDMARDs: Biologic disease modifying anti-rheumatic drugs
PROs: Patient-reported outcomes
VAS: Visual Analogue Scale
VAS-pain: Visual Analogue Scale of pain
VAS-GH: Visual Analogue Scale global health
VAS-PH: Visual Analogue Scale Physician Health
HAQ-S: Assessment Questionnaires modified for spondyloarthritis
BASFI: Bath Ankylosing Spondylitis Functional Index
BASDAI: Bath Ankylosing Spondylitis disease activity index
PASI: Psoriasis Area and Severity Index
DAPSA: Disease Activity in Psoriatic Arthritis
ASDAS: Ankylosing Spondylitis Disease Activity Score
LEI: Leeds Enthesitis Index
ESR: Erythrocyte sedimentation rate
CRP: C-reactive protein
MDA: Minimal disease activity
REM: Remission
LDA: Low
MoDA: Moderate
HAD: High
IQR: Interquartile range
DIP: Distal interphalangeal joint
T/SJ: Tender/swollen joints
IBD: Inflammatory bowel disease
MetS: Metabolic syndrome
PASDAS: Psoriatic Arthritis Disease Activity Score

## References

[1] Gladman D, Antoni C, Mease P, Clegg D, Nash P. Psoriatic arthritis: epidemiology, clinical features, course, and outcome. Ann Rheum Dis. 2005;64(Suppl 2):ii14–7. 10.1136/ard.2004.032482. 15708927 10.1136/ard.2004.032482 PMC1766874

[2] Gladman D. Early psoriatic arthritis. Rheum Dis Clin North Am. 2012;38(2):373–86. 10.1016/j.rdc.2012.05.005. 22819090 10.1016/j.rdc.2012.05.005

[3] Mease PJ, Armstrong AW. Managing patients with psoriatic disease: the diagnosis and pharmacologic treatment of psoriatic arthritis in patients with psoriasis. Drugs. 2014;74:423–41. 10.1007/s40265-014-0191-y. 24566842 10.1007/s40265-014-0191-y PMC3958815

[4] Denis A, Sztejkowski C, Arnaud L, Becker G, Felten R. The 2023 pipeline of disease-modifying antirheumatic drugs (DMARDs) in clinical development for spondyloarthritis (including psoriatic arthritis): a systematic review of trials. RMD Open. 2023;9(3): e003279. 10.1136/rmdopen-2023-003279. 37507210 10.1136/rmdopen-2023-003279 PMC10387652

[5] Colombo D, Frassi M, Pagano Mariano G, the CHRONOS Study Group. Real-world evidence of biologic treatments in psoriatic arthritis in Italy: results of the CHRONOS (EffeCtiveness of biologic treatments for psoriatic artHRitis in Italy: an ObservatioNal lOngitudinal Study of real-life clinical practice) observational longitudinal study. BMC Rheumatol. 2022;6(1):57. 10.1186/s41927-022-00284-w. 36089612 10.1186/s41927-022-00284-w PMC9464489

[6] Gossec L, Baraliakos X, Kerschbaumer A, et al. EULAR recommendations for the management of psoriatic arthritis with pharmacological therapies: 2019 update. Ann Rheum Dis. 2020;79(6):700–12. 10.1136/annrheumdis-2020-217159. 32434812 10.1136/annrheumdis-2020-217159 PMC7286048

[7] Coates LC, Soriano ER, Corp N, et al. GRAPPA Treatment recommendations domain subcommittees Group for Research and Assessment of Psoriasis and Psoriatic Arthritis (GRAPPA): updated treatment recommendations for psoriatic arthritis 2021. Nat Rev Rheumatol. 2022;18(8):465–79. 10.1038/s41584-022-00798-0. 35761070 10.1038/s41584-022-00798-0 PMC9244095

[8] Marchesoni A, Olivieri I, Salvarani C, et al. Recommendations for the use of biologics and other novel drugs in the treatment of psoriatic arthritis: 2017 update from the Italian society of Rheumatology. Clin Exp Rheumatol. 2017;35(6):991–1010. 29185959

[9] Langley RG, Elewski BE, Lebwohl M, ERASURE Study Group; FIXTURE Study Group, et al. Secukinumab in plaque psoriasis–results of two phase 3 trials. N Engl J Med. 2014;371(4):326–38. 10.1056/NEJMoa1314258. 25007392 10.1056/NEJMoa1314258

[10] McInnes IB, Mease PJ, Kirkham B, et al. FUTURE 2 Study Group. Secukinumab, a human anti-interleukin-17A monoclonal antibody, in patients with psoriatic arthritis (FUTURE 2): a randomised, double-blind, placebo-controlled, phase 3 trial. Lancet. 2015;386(9999):1137–46. 10.1016/S0140-6736(15)61134-5. 26135703 10.1016/S0140-6736(15)61134-5

[11] Baeten D, Sieper J, Braun J, MEASURE 1 Study Group; MEASURE 2 Study Group, et al. Secukinumab, an Interleukin-17A inhibitor, in Ankylosing Spondylitis. N Engl J Med. 2015;373(26):2534–48. 10.1056/NEJMoa1505066. 26699169 10.1056/NEJMoa1505066

[12] Mease PJ, Kavanaugh A, Reimold A, et al. FUTURE 1 study group. Secukinumab provides sustained improvements in the signs and symptoms of psoriatic arthritis: final 5-year results from the phase 3 FUTURE 1 study. ACR Open Rheumatol. 2020;2(1):18–25. 10.1002/acr2.11097. 31943974 10.1002/acr2.11097 PMC6957920

[13] McInnes IB, Behrens F, Mease PJ, EXCEED Study Group, et al. Secukinumab versus adalimumab for treatment of active psoriatic arthritis (EXCEED): a double-blind, parallel- group, randomised, active-controlled, phase 3b trial. Lancet. 2020;395(10235):1496–505. 10.1016/S0140-6736(20)30564-X. 32386593 10.1016/S0140-6736(20)30564-X

[14] Coates LC, Mease PJ, Gladman DD, Navarra S, Bao W, Gaillez C. Secukinumab improves physical function and quality of life and inhibits structural damage in patients with PsA with sustained remission or low disease activity: results from the 2-year phase 3 FUTURE 5 study. RMD Open. 2023;9(2):e002939. 10.1136/rmdopen-2022-002939. 37094983 10.1136/rmdopen-2022-002939 PMC10124319

[15] Deodhar A, Mease PJ, McInnes IB, et al. Long-term safety of secukinumab in patients with moderate-to-severe plaque psoriasis, psoriatic arthritis, and ankylosing spondylitis: integrated pooled clinical trial and post-marketing surveillance data. Arthritis Res Ther. 2019;21(1):111. 10.1186/s13075-019-1882-2. 31046809 10.1186/s13075-019-1882-2 PMC6498580

[16] Mease P, van der Heijde D, Landewé R, et al. Secukinumab improves active psoriatic arthritis symptoms and inhibits radiographic progression: primary results from the randomised, double-blind, phase III FUTURE 5 study. Ann Rheum Dis. 2018;77(6):890–7. 10.1136/annrheumdis-2017-212687. 29550766 10.1136/annrheumdis-2017-212687 PMC5965348

[17] Nash P, Mease PJ, McInnes IB, et al. FUTURE 3 study group. Efficacy and safety of secukinumab administration by autoinjector in patients with psoriatic arthritis: results from a randomized, placebo-controlled trial (FUTURE 3). Arthritis Res Ther. 2018;20(1):47. 10.1186/s13075-018-1551-x. 29544534 10.1186/s13075-018-1551-x PMC5856314

[18] Shirley M, Scott LJ. Secukinumab: a review in psoriatic arthritis. Drugs. 2016;76:1135–45. 10.1007/s40265-016-0602-3. 27299434 10.1007/s40265-016-0602-3

[19] Blonde L, Khunti K, Harris SB, Meizinger C, Skolnik NS. Interpretation and impact of real-world Clinical Data for the practicing clinician. Adv Ther. 2018;35(11):1763–74. 10.1007/s12325-018-0805-y. 30357570 10.1007/s12325-018-0805-y PMC6223979

[20] Fortin M, Dionne J, Pinho G, Gignac J, Almirall J, Lapointe L. Randomized controlled trials: do they have external validity for patients with multiple comorbidities? Ann Fam Med. 2006;4(2):104–8. 10.1370/afm.516. 16569712 10.1370/afm.516 PMC1467012

[21] Chimenti MS, Fonti GL, Conigliaro P, et al. One-year effectiveness, retention rate, and safety of secukinumab in ankylosing spondylitis and psoriatic arthritis: a real-life multicenter study. Expert Opin Biol Ther. 2020;20(7):813–21. 10.1080/14712598.2020.1761957. 32401062 10.1080/14712598.2020.1761957

[22] Ramonda R, Lorenzin M, Sole Chimenti M, et al. Effectiveness and safety of secukinumab in axial spondyloarthritis: a 24-month prospective, multicenter real-life study. Ther Adv Musculoskelet Dis. 2022;14:1759720X221090310. 10.1177/1759720X221090310. 35510168 10.1177/1759720X221090310 PMC9058366

[23] Dastoli S, Passante M, Loconsole F, et al. Long-term efficacy and safety of secukinumab in real life: a 240 weeks multicenter study from Southern Italy. J Dermatolog Treat. 2023;34(1):2200868. 10.1080/09546634.2023.2200868. 37026590 10.1080/09546634.2023.2200868

[24] Kiltz U, Sfikakis PP, Gaffney K, et al. Secukinumab Use in patients with moderate to severe psoriasis, Psoriatic Arthritis and Ankylosing spondylitis in Real-World setting in Europe: Baseline Data from SERENA Study. Adv Ther. 2020;37(6):2865–83. 10.1007/s12325-020-01352-8. 32378070 10.1007/s12325-020-01352-8 PMC7467439

[25] Michelsen B, Georgiadis S, Di Giuseppe D, et al. Real-world six- and twelve-Month Drug Retention, Remission, and response rates of Secukinumab in 2,017 patients with psoriatic arthritis in Thirteen European Countries. Arthritis Care Res (Hoboken). 2022;74(7):1205–18. 10.1002/acr.24560. 33460531 10.1002/acr.24560

[26] Valero-Expósito M, Martín-López M, Guillén-Astete C, et al. Retention rate of secukinumab in psoriatic arthritis: real-world data results from a Spanish multicenter cohort. Med (Baltim). 2022;101(36):e30444. 10.1097/MD.0000000000030444. 10.1097/MD.0000000000030444 PMC10980406 36086678

[27] Glintborg B, Ostergaard M, Krogh NS, et al. Clinical response, drug survival, and predictors thereof among 548 patients with psoriatic arthritis who switched tumor necrosis factor α inhibitor therapy: results from the Danish Nationwide DANBIO Registry. Arthritis Rheum. 2013;65(5):1213–23. 10.1002/art.37876. 23460467 10.1002/art.37876

[28] Lorenzin M, Ortolan A, Cozzi G, et al. Predictive factors for switching in patients with psoriatic arthritis undergoing anti-TNFα, anti-IL12/23, or anti-IL17 drugs: a 15-year monocentric real-life study. Clin Rheumatol. 2021;40(11):4569–80. 10.1007/s10067-021-05799-0. 34136971 10.1007/s10067-021-05799-0 PMC8519923

[29] Cañete JD, Tasende JAP, Laserna FJR, Castro SG, Queiro R. The impact of comorbidity on patient-reported outcomes in psoriatic arthritis: a systematic literature review. Rheumatol Ther. 2020;7:237–57. 10.1007/s40744-020-00202-x. 32270447 10.1007/s40744-020-00202-x PMC7211228

[30] Scagnellato L, Collesei A, Doria A, GISEA Study Group, et al. Comorbidities in the Spondyloarthritis GISEA cohort: an average treatment effect analysis on patients treated with bDMARDs. Clin Exp Rheumatol. 2023;30. 10.55563/clinexprheumatol/q38lu0. 10.55563/clinexprheumatol/q38lu0 37650298

[31] Ruscitti P, Pantano I, Perrotta FM, et al. The assessment of the drug retention rate of secukinumab in patients with psoriatic arthritis in a real-life multicentre cohort. Clin Exp Rheumatol. 2023;24. 10.55563/clinexprheumatol/tpp63h. 10.55563/clinexprheumatol/tpp63h 37497733

[32] García-Dorta A, León-Suarez P, Peña S, et al. Association of Gender, diagnosis, and obesity with Retention Rate of Secukinumab in Spondyloarthropathies: results form a Multicenter Real-World Study. Front Med (Lausanne). 2022;8: 815881. 10.3389/fmed.2021.815881. 35096907 10.3389/fmed.2021.815881 PMC8792854

[33] Alonso S, Villa I, Fernández S, et al. Multicenter Study of Secukinumab Survival and Safety in Spondyloarthritis and Psoriatic Arthritis: SEcukinumab in Cantabria and ASTURias Study. Front Med (Lausanne). 2021;8: 679009. 10.3389/fmed.2021.679009. 34124110 10.3389/fmed.2021.679009 PMC8187784

[34] Taylor W, Gladman D, Helliwell P, Marchesoni A, Mease P, Mielants H, CASPAR Study Group. Classification criteria for psoriatic arthritis: development of new criteria from a large international study. Arthritis Rheum. 2006;54(8):2665–73. 10.1002/art.21972. 16871531 10.1002/art.21972

[35] Secukinumab. Summary of product characteristics. Available from: http://www.ema.europa.eu/index.jsp?curl=pages/medicines/human/medicines/003729/human_med_001832.jsp&mid=WC0b01ac058001d124cited. Cited 2019. Apr 25.

[36] Mease PJ. Measures of psoriatic arthritis: Tender and Swollen Joint Assessment, Psoriasis Area and Severity Index (PASI), Nail Psoriasis Severity Index (NAPSI), Modified Nail Psoriasis Severity Index (mNAPSI), Mander/Newcastle Enthesitis Index (MEI), Leeds Enthesitis Index (LEI), Spondyloarthritis Research Consortium of Canada (SPARCC), Maastricht Ankylosing Spondylitis Enthesis Score (MASES), Leeds Dactylitis Index (LDI), Patient Global for Psoriatic Arthritis, Dermatology Life Quality Index (DLQI), Psoriatic Arthritis Quality of Life (PsAQOL), Functional Assessment of Chronic Illness Therapy-Fatigue (FACIT-F), Psoriatic Arthritis Response Criteria (PsARC), Psoriatic Arthritis Joint Activity Index (PsAJAI), Disease Activity in Psoriatic Arthritis (DAPSA), and Composite Psoriatic Disease Activity Index (CPDAI). Arthritis Care Res (Hoboken). 2011;63 Suppl 11:S64-85. 10.1002/acr.20577. 22588772 10.1002/acr.20577

[37] Coates LC, Helliwell PS. Validation of minimal disease activity criteria for psoriatic arthritis using interventional trial data. Arthritis Care Res (Hoboken). 2010;62(7):965–9. 10.1002/acr.20155. 20589696 10.1002/acr.20155

[38] Schoels MM, Aletaha D, Alasti F, Smolen JS. Disease activity in psoriatic arthritis (PsA): defining remission and treatment success using the DAPSA score. Ann Rheum Dis. 2016;75(5):811–8. 10.1136/annrheumdis-2015-207507. 26269398 10.1136/annrheumdis-2015-207507

[39] Glintborg B, Di Giuseppe D, Wallman JK, et al. Uptake and effectiveness of newer biologic and targeted synthetic disease-modifying antirheumatic drugs in psoriatic arthritis: results from five nordic biologics registries. Ann Rheum Dis. 2023;82(6):820–8. 10.1136/ard-2022-223650. 36813538 10.1136/ard-2022-223650

[40] Fagerli KM, Lie E, van der Heijde D, et al. Switching between TNF inhibitors in psoriatic arthritis: data from the NOR-DMARD study. Ann Rheum Dis. 2013;72:1840–4. 10.1136/annrheumdis-2012-203018. 23562987 10.1136/annrheumdis-2012-203018

[41] Orbai A-M, McInnes IB, Coates LC, et al. Effect of Secukinumab on the different GRAPPA-OMERACT core domains in psoriatic arthritis: a pooled analysis of 2049 patients. J Rheumatol. 2020;47:854–64. 10.3899/jrheum.190507. 31615919 10.3899/jrheum.190507

[42] Coates LC, Gladman DD, Nash P, et al. FUTURE 2 study group. Secukinumab provides sustained PASDAS-defined remission in psoriatic arthritis and improves health-related quality of life in patients achieving remission: 2-year results from the phase III FUTURE 2 study. Arthritis Res Ther. 2018;20(1):272. 10.1186/s13075-018-1773-y. 30526678 10.1186/s13075-018-1773-y PMC6286532

[43] Moreno-Ramos MJ, Sanchez-Piedra C, Martínez-González O, et al. Real-world effectiveness and Treatment Retention of Secukinumab in patients with psoriatic arthritis and Axial spondyloarthritis: a descriptive observational analysis of the Spanish BIOBADASER Registry. Rheumatol Ther. 2022;9(4):1031–47. 10.1007/s40744-022-00446-9. 35467242 10.1007/s40744-022-00446-9 PMC9314517

[44] Ramonda R, Puato M, Punzi L, et al. Atherosclerosis progression in psoriatic arthritis patients despite the treatment with tumor necrosis factor-alpha blockers: a two-year prospective observational study. Joint Bone Spine. 2014;81(5):421–5. 10.1016/j.jbspin.2014.02.005. 24703399 10.1016/j.jbspin.2014.02.005

[45] Kim JH, Choi IA. Cardiovascular morbidity and mortality in patients with spondyloarthritis: a me-ta-analysis. Int J Rheum Dis. 2021;24(4):477–86. 10.1111/1756-185X.13970. 32969177 10.1111/1756-185X.13970

[46] Ortolan A, Lorenzin M, Felicetti M, Ramonda R. Do obesity and overweight influence Disease Activity measures in Axial Spondyloarthritis? A systematic review and Meta-analysis. Arthritis Care Res. 2021;73(12):1815–25. 10.1002/acr.24416. 10.1002/acr.24416 32799405

[47] Pantano I, Iacono D, Favalli EG, et al. Secukinumab efficacy in patients with PsA is not dependent on patients’ body mass index. Ann Rheum Dis. 2022;81(3):e42. 10.1136/annrheumdis-2020-217251. 32169970 10.1136/annrheumdis-2020-217251

[48] von Stebut E, Boehncke WH, Ghoreschi K, et al. IL-17A in Psoriasis and Beyond: Cardiovascular and metabolic implications. Front Immunol. 2020;10: 3096. 10.3389/fimmu.2019.03096. 32010143 10.3389/fimmu.2019.03096 PMC6974482

[49] Ortolan A, Lorenzin M, Leo G, et al. Secukinumab Drug Survival in Psoriasis and Psoriatic Arthritis patients: a 24-Month Real-Life Study. Dermatology. 2022;238(5):897–903. 10.1159/000522008. 35263743 10.1159/000522008

[50] Baraliakos X, Gossec L, Pournara E, et al. Secukinumab in patients with psoriatic arthritis and axial manifestations: results from the double-blind, randomised, phase 3 MAXIMISE trial. Ann Rheum Dis. 2021;80(5):582–90. 10.1136/annrheumdis-2020-218808. 33334727 10.1136/annrheumdis-2020-218808 PMC8053347

[51] Adami G, Idolazzi L, Benini C, et al. Secukinumab retention rate is greater in patients with psoriatic arthritis presenting with axial involvement. Reumatismo. 2023;75(1). 10.4081/reumatismo.2023.1559. 10.4081/reumatismo.2023.1559 37154254

[52] Fassio A, Atzeni F, Rossini M, On Behalf Of The Study Group On Osteoporosis And Skeletal Metabolic Diseases Of The Italian Society Of Rheumatology, et al. Osteoimmunology of Spondyloarthritis. Int J Mol Sci. 2023;24(19): 14924. 10.3390/ijms241914924. 37834372 10.3390/ijms241914924 PMC10573470

[53] Fassio A, Gatti D, Rossini M, et al. Secukinumab produces a quick increase in WNT signalling antagonists in patients with psoriatic arthritis. Clin Exp Rheumatol. 2019;37(1):133–6. 30418122

[54] Braun J, Buehring B, Baraliakos X, et al. Effects of Secukinumab on Bone Mineral density and bone turnover biomarkers in patients with Ankylosing spondylitis: 2-Year Data from a phase 3 study, MEASURE 1. BMC Musculoskelet Disord. 2021;22:1037. 10.1186/s12891-021-04930-1. 34903218 10.1186/s12891-021-04930-1 PMC8670267

[55] Schreiber S, Colombel J-F, Feagan BG, et al. Incidence rates of inflammatory bowel disease in patients with psoriasis, psoriatic arthritis and ankylosing spondylitis treated with secukinumab: a retrospective analysis of pooled data from 21 clinical trials. Ann Rheum Dis. 2019;78:473–9. 10.1136/annrheumdis-2018-214273. 30674475 10.1136/annrheumdis-2018-214273 PMC6530077

