# Supplementary material for: Four-year effectiveness, safety and drug retention rate of secukinumab in psoriatic arthritis: a real-life Italian multicenter cohort
Source: Arthritis Res Ther. 2024 Sep 28;26:172. doi: 10.1186/s13075-024-03401-x (PMC11438205; doi:10.1186/s13075-024-03401-x)
Supplement: Supplementary file 1 — Supplementary Material 1. [file 13075_2024_3401_MOESM1_ESM.docx]

**Supplementary Table 1. Clinical, functional, disease activity, and serological parameters of all (n = 685) PsA patients during the 48-month follow-up period.**

| **Total 685 PsA patients** | **T0** | **T6** | **T12** | **T24** | **T36** | **T48** | ***p*** |
| --- | --- | --- | --- | --- | --- | --- | --- |
| **TJ [0-68], median (IQR)** | 6 (2-11) | 2 (0-5) | 2 (0-4) | 0 (0-2) | 0 (0-2) | 0 (0-1) | <0.01 |
| **SJ [0-66], median (IQR)** | 1 (0-3) | 1 (0-2) | 1 (0-1) | 0 (0-1) | 0 (0-0) | 0 (0-0) | 0.03 |
| **LEI [0-6], mean (SD)** | 2 (0-3) | 1 (0-2) | 1 (0-1) | 0.5 (0-1) | 0 (0-0.5) | 0 (0-0) | 0.04 |
| **Dactylitis [0-20], median (IQR)** | 0.7 (0.4-0.9) | 0.7 (0.4-1.5) | 0.2 (0.1-0.9) | 0 (0-0) | 0 (0-0) | 0 (0-0) | 0.05 |
| **PASI [0-72], median (IQR)** | 3.2 (1.2-5.6) | 0.5 (0.0-3.5) | 0.2 (0.0-2.4) | 0.1 (0.0-2.1) | 0.1 (0.0-1.3) | 0.0 (0.0-0.5) | <0.01 |
| **ESR [0-25](mm/h), median (IQR)** | 15 (7-27) | 11 (6.0-21.5) | 11 (5-19) | 10 (5-18) | 11 (6-17) | 9 (5-15) | 0.04 |
| **CRP [0-6](mg/L), median (IQR)** | 3.3 (1.3-7.9) | 2.9 (1.1-5.0) | 2.1 (1.0-4.8) | 2.0 (1.0-4.0) | 2.0 (1.0-4.0) | 2.0 (1.0-3.7) | 0.03 |
| **DAPSA [0-164], median (IQR)** | 23.6 (17.1-30.5) | 12.2 (8.0-19.9) | 10 (5.1-16.0) | 7 (2.2-13.0) | 5.1 (2.0-12.0) | 4.2 (2.0-10.0) | <0.01 |
| **ASDAS [0-6], median (IQR)** | 3.1 (2.3-3.6) | 2.18 (1.3-2.9) | 1.8 (1.1-2.7) | 1.5 (0.9-2.1) | 1.4 (0.7-2.1) | 1.2 (0.6-2.0) | 0.02 |
| **HAQ-S [0-8], median (IQR)** | 1.3 (0.9-1.8) | 1 (0.4-1.3) | 0.6 (0.1-1.0) | 0.4 (0.1-0.9) | 0.3 (0.0-0.6) | 0.1 (0.0-0.5) | 0.05 |
| **VAS-pain [0-10], median (IQR)** | 7 (6-8) | 5 (3-7) | 4 (2-6) | 3 (1-5) | 2 (1-5) | 2 (1-4) | 0.02 |
| **VAS-GH [0-10], median (IQR)** | 7 (5-8) | 5 (3-6) | 4 (2-6) | 3 (1-5.7) | 3 (1-5) | 2 (1-5) | 0.02 |
| **VAS-PH [0-10], median (IQR)** | 7 (5-7) | 4 (2-5) | 2.5 (1-5) | 2.1 (1-4) | 1 (0-4) | 1 (0-3) | 0.03 |
| **BASDAI [0-10], median (IQR)** | 5.5 (4.2-6.9) | 3.5 (2.0-5.2) | 2.6 (1.5-4.3) | 2.1 (0.8-3.3) | 1.5 (0.2-3.0) | 1.1 (0.1-2.7) | 0.03 |
| **BASFI [0-10], median (IQR)** | 6 (4.4-7.0) | 4 (2.5-5.2) | 2.7 (1.7-4.1) | 1.8 (0.4-3.0) | 1.3 (0.7-2.2) | 1.1 (0.1-2.0) | 0.03 |

Data are expressed as median (interquartile range [IQR]). Values were computed by chi-square test (for proportion) or the Wilcoxon test (for continuous data) *p* ≤ *0.05* T24 vs. T0.

**Legend**: TJ, Tender Joint; SJ, Swollen Joint; LEI, Leeds Enthesitis Index; PASI, Psoriasis Area Severity Index; ESR, erythrocyte sedimentation rate; CRP, C-reactive protein; DAPSA, Disease Activity Index for Psoriatic Arthritis; ASDAS, Ankylosing Spondylitis Disease Activity Score; HAQ-S: Health Assessment Questionnaire modified for spondyloarthritis; VAS-pain, Visual Analogue Scale-pain; VAS-GH, Visual Analogue Scale global health; BASDAI: Bath Ankylosing Spondylitis Disease Activity Index; BASFI, Bath Ankylosing Spondylitis Functional Index.

**Supplementary Table 2. Comorbidities of naïve (n = 225) and biological agents failure (n = 460) PsA patients.**

|  |  |  |  |  |
| --- | --- | --- | --- | --- |
| **Comorbidities** | **total patients** | **naïve** | **non-naïve** | ***p§*** |
| **metabolic syndrome (MetS)** | 75 (10.9%) | 11 (4.9%) | 64 (13.9%) | *0.03* |
| **hypertension** | 245 (35.8%) | 60 (26.7%) | 185 (40.2%) | *0.04* |
| **ischemic heart disease** | 53 (7.7%) | 11 (4.9%) | 42 (9.1%) | *0.04* |
| **dyslipidemia** | 191 (27.9%) | 53 (23.6%) | 138 (30.0%) | *0.03* |
| **hyperuricemia** | 73 (10.7%) | 17 (7.6%) | 56 (12.2%) | *0.04* |
| **type II diabetes** | 67 (9.8%) | 20 (8.9%) | 47 (10.2%) | *0.05* |
| **gastritis, gastric ulcer or dyspeptic disorders** | 61 (8.9%) | 18 (8.0%) | 43 (9.3%) | *ns* |
| **liver disease (e.g. steatosis)** | 75 (10.9%) | 14 (6.2%) | 61 (13.3%) | *0.03* |
| **pneumopathies** | 42 (6.1%) | 6 (2.7%) | 36 (7.8%) | *0.02* |
| **thyroid disorder** | 84 (12.3%) | 25 (11.1%) | 59 (12.8%) | *ns* |
| **osteoporosis** | 68 (9.9%) | 22 (9.8%) | 46 (10.0%) | *ns* |
| **kidney failure** | 14 (2.1%) | 5 (2.2%) | 9 (1.9%) | *ns* |
| **depression** | 66 (9.6%) | 16 (7.1%) | 50 (10.9%) | *0.04* |
| **fibromyalgia** | 131 (19.1%) | 31 (13.8%) | 100 (21.7%) | *0.04* |
| **neurological disorders (such as neuropathy)** | 40 (5.8%) | 5 (2.2%) | 35 (7.6%) | *0.05* |
| **positive Mantoux TB skin test or QuantiFERON TB Gold test** | 54 (7.9%) | 18 (8.0%) | 36 (7.8%) | *ns* |
| **previous hepatitis B** | 33 (4.8%) | 12 (5.3%) | 21 (4.6%) | *ns* |
| **previous hepatitis C** | 15 (2.2%) | 5 (2.2%) | 10 (2.2%) | *ns* |
| **previously eradicated cancer** | 44 (6.4%) | 11 (4.9%) | 33 (7.2%) | *0.05* |

**Legend:** Values are expressed as frequencies (absolute number and percentage). p ≤ 0.05. ns: not statistically significant. Values were computed by chi-square test (for proportion). naïve: naïve to bDMARDs; non-naïve: bDMARDs failure.
